# Road traffic accident and management outcome among in Adama Hospital Medical College, Central Ethiopia

**DOI:** 10.11604/pamj.2021.38.190.11650

**Published:** 2021-02-19

**Authors:** Eskindir Deresse, Meyrema Abdo Komicha, Tesfaye Lema, Shemsedin Abdulkadir, Kedir Teji Roba

**Affiliations:** 1 Department of Public Health, Adama Hospital Medical College, Shewa, South Eastern Ethiopia,; 2 School of Nursing and Midwifery, College of Health and Medical Science, Haramaya University, Harar, Ethiopia

**Keywords:** Road traffic injury, management, outcome, Ethiopia

## Abstract

**Introduction:**

road traffic injuries represent accounts for significant cause of morbidity and mortality around the globe, particularly in developing countries like Ethiopia. Poor pre-hospital care system and delays in hospitals before getting aids added to the woes of mortality. However, there are no study that determine the types of injury, management and outcome of road traffic accidents and associated factors in this study area.

**Methods:**

a hospital based retrospective cross sectional study was conducted among patients attending to Adama Hospital Medical College with accidental injuries from January to December 2015. Data were retrieved from 556 patients registry selected by systematic random sampling from 11,120 injuries visiting the hospital. Data were coded, cleaned and entered to SPSS version 20 for analysis. Factors associated with the management outcome of injury related to road traffic accident were analyzed and statistical significance was declared with p < 0.05 with CI of 95%.

**Results:**

out of 556 trauma victims, 304 (54.7%) were due to road traffic accidents followed by personal violence (24%) and falling accident (10.3%). The majorities (74.8%) of patients were male and urban residents (55%). Soft tissue injury was the most frequent type of injury (51%) followed by extremity fracture and dislocation (26%). Delay to come to hospital (over 24 hours), severity of injuries and management types were factors influencing management outcome of injuries related to traffic accidents. About 90.1%, 4.6% and 5.3% of the patients were discharged without any prominent disability, permanent disability and died respectively.

**Conclusion:**

road traffic accidents are preventable causes of morbidity and mortality. Practices of strict road safety measures and appropriate use of roadways by pedestrians should be in place, while establishing and strengthening early access to hospital and pre-hospital care to save life of injuries.

## Introduction

Accidental injuries represent a major cause of morbidity and mortality in around the globe [1]. The Global Burden of Disease (GBD) study estimates that 10% of global deaths are due to accidental injuries [2]. Globally, Road Traffic Accidents (RTAs) are common types of injuries and death that is major public health problems, especially in developing countries [3]. In 2013, World Health Organization (WHO) indicated that more than 1.24 million people die every year as a result of RTAs, making it the eighth leading cause of death globally, and the leading cause of death for young people aged 15-29. Based on this trends, it is projected to be the fifth leading cause of death globally by 2030 [4]. In the developing world, the improved life expectancy together with industrialization and urbanization are putting heavy pressure on the transport system in general and on the road system in particular. In Africa a retrospective analysis of non-fatal road traffic crush victims still showed that the commonest injuries were fractures (69.0%) with the tibia/fibula being most fractured bones (30.3%) [5].

Study conducted in Jima reveled that fracture was the leading outcome of injury (41.2%), followed by bruise or laceration (36.7%), internal organ injury (9%) and sprain or/and dislocation 5.1%. With regard to injury spectrum, 32.7%, 41.3%, 26% were classified as having had severe, moderate, minor injuries respectively [6]. An others community based study in Jimma Zone indicated a magnitude of 8.9% [7]. Studies in Ethiopia found that males and young adults aged below 40 years were the most vulnerable groups to injury [7, 8]. A study in Tikur Anbessa Hospital indicated that 77% of cases of RTA were unintentional injuries [8].

A study in Ethio-Swedish children's Hospital in Ethiopia indicated that accidents and trauma accounted for 25% surgical admissions with the commonest conditions were burns, car accidents, accidental falls, and foreign body aspirations (13). While a study in Tikur Anbessa Hospital showed motor vehicle injuries accounted for 41% of all causes and accidental fall and interpersonal assault accounted for 21% and 20%, respectively. The overall admitted rate due to injuries cases were 11.6% with an overall mortality of 1.47% (11). Another study done in north Gondar indicated that the leading causes of injury were assault (48.5%), fall (18.6%) and road traffic (14.7%) (8). According a study in Jimma Zone, the most common causes of injury were cut by sharp tools (33.5%), falling (20.9%) and stab (17.6%). To the knowledge of these researchers, there is no study conducted in central part of Ethiopia, particularity Adama Hospital and Medical College Adama The second largest city of Ethiopia. This study was aimed at assessing the prevalence of road traffic injury and treatment out comes among patients attending emergency outpatient department of Adama Hospital and Medical College due to different injuries.

## Methods

### Study area and period

The study was conducted in Adama Hospital Medical College that is located 100 km away from Addis Ababa (the capital city of Ethiopia) on South East direction. The hospital has pediatric and adult surgical wards in addition to orthopedic ward that have all together 53 beds. First and second year surgical resident student and first to third year Integrated Emergency Surgical Officers students cover the emergency surgical OPD and wards, consulted by two orthopedic surgeons and four general surgeons. Currently the hospital is giving a referral services for its catchment area for more than six million population. It receives patients on referral mainly from East Shewa Zone, West Hararghe Zone, Afar and Ahmara region. This study was conducted from May to July 30, 2016 from available data in hospital from January to December 2015 in Adama hospital medical college.

### Study design

Hospital based cross-sectional study design was employed with record reviews of one year data (from January to December 2015) of injured patients who visited and managed at EOPD, surgical and orthopedic wards of Adama Hospital Medical College (AHMC).

### Study population and sample size

The study was conducted on records or charts of all injured patients with complete information or records who was treated at Emergency outpatient department (EOPD), surgical and orthopedic wards of the hospital during the period of January to December 2015. Patients died on arrival to EOPD, referred cases to other facilities with unknown treatment out comes were excluded. Single population proportion formula was used to calculate sample size by considering the population proportion 50% (no previous studies) with 95% confidence interval 20 % incomplete data rate. Therefore total sample size was 556 that included in the study.

### Sampling procedure

During one year study period, a total of 11,120 trauma cases were treated in AHMC. The study subjects were selected using simple random sampling technique by taking discharge book of surgical, orthopedic ward and EOPD. By systematic random sampling methods employed using 11,120 cases as a sampling frame, data were reviewed from charts from an interval of every 20 cards after taking the first sample by lottery method.

### Data collection and quality management

The data was collected by using structured checklist prepared in English. The checklist contains the following main categories of variables. Socio demographic characteristics, cause of injury, type of car, site of injury, type of injury or diagnosis and outcome. Three first year IESO students were recruited as data collectors and two second year students were selected as supervisors. Training was given for the data collectors and supervisors before data collection. During the data collection, record keepers were sort out all the trauma cases from log books and medical records. Data collectors traced and collected data from randomly identified charts of cases using checklist. The quality of the data was assured through careful design, careful preparation of checklists, Proper training and close supervision of data collectors, proper handling of data and frequent monitoring of activities. All collected data were examined for completeness and consistency at the end of each day.

### Data processing and analysis

The collected data were cleaned, coded and entered in to SPSS version 20 for analysis. Descriptive statistical methods were used to generate frequencies, proportion and the result presented using table and graphs. Bivariate analysis was employed to identify the explanatory variables having association with the outcome variable and qualified for the final model. Each variable with statistically significant in bivariate analysis entered in to multiple logistic regression model as the independent variable and management outcome of RTI status being a dependent variable. The presence and strength of association was measured by adjusted odd ratio (95% CI). P ≤ 0.05 considered as statistically significant for all the independent variables in the final model.

### Ethical consideration

Ethical clearance was obtained from the ethical review board of Adama Hospital Medical College. The advantages and purposes of the study explained to staff members of the hospitals. Then, for retrieval of individual record and confidentiality of information, a written consent was given to the record office of the hospitals.

## Results

### Socio demographic characteristics

A total of 556 trauma victims registry were included in the study. Of all victims visited the hospitals 74.8% were male and 25.2% were female. The mean age of the victims was 29.05, ranging from one to ninety five years old. The most commonly affected age ranges were between 20-29 years old (36.3%), followed by age 30-39 years (17.3%). Majority of the victims were from rural areas as depicted on Table 1.

**Table 1:** socio demographic characteristics of trauma victim patients who visited AHMC from January 1 to December 2015, Adama, Ethiopia

Characteristics of patients		Road traffic accident	Non road traffic accident	Total
		N (%)	N (%)	N (%)
Age	<20	64 (21.1)	71 (28.2)	135 (24.3)
	20-29	107 (35.2)	95 (37.7)	202 (36.3)
	30-39	63 (20.7)	34 (13.5)	97 (17.5)
	≥40	70 (23.0)	52(20.7)	122 (21.9)
Sex	Male	239(78.6)	177 (70.2)	416 (74.8)
	Female	65 (21.4)	75 (29.8)	140 (25.2)
Area of residence	Urban	168 (55.3)	137 (54.4)	305 (55)
	Rural	136 (44.7)	115 (45.6)	251 (45)

### Causes of injury and delay in pre-hospital phases

Among 554 cases reviewed, road traffic accidents were the leading cause of injury, accounting for 54.7% of all trauma victims followed by Personal violence 24%. Even though majority (80.6%) of the patient came to hospital within 24 hours of accidents, the rest of them visited the hospital after one day of injury. The majority of the victims were pedestrians which accounts for 94 people (71.7%), followed by passengers which consist of 17 people (13%) and drivers which constitute 16 people (12.2%) (Table 2).

**Table 2:** causes of injury and type of vehicle that causes RTIs among trauma victims from January 1 to December 31, 2015, at AHMC, Adama, Ethiopia

Variable	Category	Frequency	Percent
**Cause of injury**	Road traffic accident	304	54.7
	Falling accident	57	10.3
	Personal violence	133	24
	Gun shot	10	1.7
	Burn	13	2.3
	Dog bite	22	4
	Snake bite	9	1.6
	Other animal related injury*	8	1.4
Type of injury	Soft tissue injury	155	51.0
	Fracture and dislocation	79	26.0
	Head and spine injury	27	8.9
	Torso injuries	36	11.8
	Other injuries	7	2.3
Severity of injury	Mild	129	42.4
	Moderate		34.9
	Sever		22.7
**Type of management**	Wound debridement		16.8
	Treatment of fracture and dislocation		23.0
	Limp amputation		2.6
	Suturing of wound site		35.9
	Others		
**Services provided**	Admitted to hospital	220	72.3
	Get outpatient services	84	27.6
**Duration of stay in hospital**	<week	136	44.7
	1-3 week	52	17.1
	>3 weeks	32	10.5
**Patient role during victim**	Passenger	126	41.4
	Pedestrians	135	44.4
	Others	43	14.2

### Injury location, type and severity road traffic accidents

The frequent locations of the injury were lower extremity (31.9%) followed by upper extremity (26.6%), head, neck, and face injuries (25). Soft tissue injury was the major regarding severity of injury, mild injury requiring some treatment was the leading outcome of the injuries constituting 42.4% of the injuries (Table 2).

### Type of management and length of hospital stay of injury related to RTA

Of 304 injured cases of RTAs, 72.3% of cases were admitted to surgical and orthopedic wards while 27.6% treated and discharged to their home from emergency outpatient department. Among admitted patients, mean length of stay in hospitals was 14.04 ranging from 1-120 days. Majority (44.7%) of the case stay in hospital for less than one week while 17.1% of them stay for one to three weeks (Table 2).

### Management outcome of injury related to RTA

Out of 304 RTIs, 90.1% of the patient were discharged with favorable management outcome (discharged without prominent disability), while 4.6% and 5.3% of them were discharged with permanent disability and died respectively.

### Factors influencing management outcome of injury related to RTAs

In bivariate analysis; age of the patient, residence, pre-hospital delay, site of injury, severity and type of management were significantly associated with management outcome of injury related to RTA. However, severity and type of management were has relationships and hence type of management were removed from the model (Table 3). In multivariable logistic regression pre-hospital delay greater than 24 hours and severity of the injuries were factors significantly associated with unfavorable management outcome. Pre-hospital duration greater than 24 hours were 13 times odds of unfavorable management out come as compare to visiting hospitals in less than 24 hours after injury (AOR= 13.3, 95% CI, 4.3, 41.7). Severe injuries were 22 times more odds of unfavorable management outcome as compare to mild and moderate injuries (AOR= 22.372; 95% CI 6.409, 78.091) (Table 3).

**Table 3:** factors influencing management outcome of injury related to RTA at AHMC, Adama, Ethiopia

Characteristics of the patient	Management outcome		COR(95% CI)	AOR(95%CI)
	Favorable outcome	Unfavorable outcome		
**Sex**	N (%)	N (%)		
Male	214(78.1)	25(83.3)	1.402(0.515, 3.818)	
Female	60(21.9)	5(16.7)	1	
**Age**				
≤30	175(63.9)	11(36.7)	1	1
>30	99(36.1)	19(60.3)	3.053 (1.396, 6.677)*	2.071(.716, 5.990)
**Residence**				
Urban	159(58)	9(30)	1	1
Rural	115(42)	21(70)	3.226 (1.425, 7.302)*	1.022(.291, 2.428)
**Time of arrival**				
≤24 hours	247(90.1)	11(36.7)	1	1
>24 hours	27(9.9)	19(63.3)	15.801 (6.807, 36.681)*	13.333(4.261, 41.719)*
**Site of injury**				
Extremities	166(60.6)	13(43.3)	1	1
Others organ	108(21.2)	17(30)	1.981(0.805, 4.878)	1.080(0.166, 7.036)
**Severity of injury**				
Mild and moderate	230(83.9)	5(116.7)	1	1
Severe	44(16.1)	25(83.3)	33.977(11.299, 102.172)*	22.372(6.409, 78.091)*

## Discussion

This study indicated that out of 556 trauma victims, 304 (54.7%) were due to road traffic accidents followed by personal violence (24%) and falling accidents (10.3%). The majorities (74.8%) of patients were male, urban resident´s majorities (55%). Soft tissue injury was the most frequent type of injury (51%), followed by extremity fracture and dislocation (26%). Delay to come to hospital for more than 24 hours, severity of injuries and management types were factors influencing management outcome of injuries related to RTAs. About 90.1% of the patients were discharged without any prominent disability, while 4.6% and 5.3% of them were discharged with permanent disability and died respectively. The most common cause of injury in this study was road accident injury. This was lower than study done in Welayta (62.5%) [9], Gondar (14.7%) [10], Jima (30.3) [6]. The difference may be due to the fact that this hospital is located at central part of Ethiopia and provides services to more tat six million people. This indicated that road traffic accident is major public health in Ethiopia that requires urgent public interventions.

In addition to this, the most commonly affected age groups were 20-29, most productive age group of the country. This shows That a large amount of sufferers are people of most economically active age group that subsequently leads an economic lost both to the family and the nation that demands policy makers interventions as soon as possible. It is found that significant proportion of the victims presented to the hospital after 24 hours of the injury up to 120 days. This delay is one of the delays that affect the treatment outcomes. This is similar to study conducted in Ghana where about half of the cases were presented within a week after the injury [3]. It is fact that accidental injuries should visit hospitals as soon as possible and get appropriate treatment as soon as possible to reduce lifelong complications.

Out of total victims of road traffic injury, large proportions of the victims (44.4%) were Pedestrian passengers followed by the passengers in the vehicles. This is in line with other studies [4, 11] where majority of traumatic victims were pedestrians followed by passenger. But this figure is lower than other African regions (56.5%) [12], Tanzania (55.4%) [4]. similarly, a study in Amhara region of Northern Ethiopia indicated that pedestrians were majority of cases and followed by passengers [13]. The predominance of pedestrian injury in this study may be a sign of low public consciousness on road use, shortage of pedestrian facilities in road design and poor practice of road safety measures by the general population in the country. The most frequent locations of the injury in this study were lower extremity followed by upper extremity. This was consistent with the others studies done in Jima [6]. Likewise, soft tissue injury was the most frequent type of injury and large proportions had extremity fracture and dislocation (26%). In contrast to this study fracture were the most conmen type of injury sustained Jima (41.2%) [6]. The frequent procedures performed to the patients were suturing of lacerated wound followed by treatment of fracture and dislocation. This is in line with study conducted in Nigeria [12].

Out of RTIs cases, 4.6% of the causalities were discharged with permanent disability that affects their long life routine activates. The mortality rate in this study was 5.3% which is almost similar study done in Wolayta (6%) [9]. This indicated the need for urgent call for intervention. In multivariable logistic regression pre-hospital duration >24 hours, severe injury and conservative management, were factors significantly associated with unfavorable management outcome in this study. This indicated delay to visit hospitals after injury increases the risk of complication and death due to heavy blood loss damage of organs.

### Study limitations

Findings in this study should be interpreted in the light of the inherent limitations of the study. We could not take more information on certain risk factors like educational status of victims because of lack of available data from the records; since this was a hospital based cross-sectional study.

## Conclusion

According to this study finding the incidence of trauma caused by RTA was the highest cause of trauma related admission (72.3%) among all injuries. The majority of them are from urban areas while patients from rural areas more commonly presented with non-RTAs trauma. One of the major cause for death or life long disability of the victims were delay for more than 24 hours to one week before seeking medical which need urgent health information dissemination, in addition to establishing and strengthen first aid care training before arrival to hospital during delay. The provision of tailored messages to all members of the community regarding knowledge and practices of road safety measures like appropriate use of pavements by pedestrians and avoiding risky driving behaviors.

### What is known about this topic

- Road traffic accident is common in Ethiopia;
- Severity of RTA is known.

### What this study adds

- Prevalence of RTA among injuries´ in hospital;
- Management outcomes among RTA is not recorded in this hospital;
- Length of delay before arrival to the hospital in the study area.
